# Involving patients and the public in research using linked confidential patient information without consent: a case study on the COloRECTal cancer data Repository (CORECT-R)

**DOI:** 10.23889/ijpds.v9i5.2804

**Published:** 2024-09-18

**Authors:** Aden Chun Hei Kwok, Amy Downing, Paul Affleck, Hannah Rossington, Matt Howard-Murray, Julliet Lwiindi, Chen-Chun Pai, Catriona Gilmour Hamilton, Eva JA Morris

**Affiliations:** 1 Big Data Institute, Li Ka Shing Centre for Health Information and Discovery, University of Oxford; 2 Applied Health Research Unit, Nuffield Department of Population Health, University of Oxford; 3 Cancer Epidemiology Group, Leeds Institute of Medical Research and Leeds Institute for Data Analytics, University of Leeds; 4 Cancer Research UK; 5 Oxford Cancer, The CRUK Oxford Centre, University of Oxford

## Objective and Approach

In 2017, the charity Cancer Research UK funded a UK Colorectal Cancer Intelligence Hub to create the COloRECTal cancer data Repository (CORECT-R). This was intended to be a single research data system that links all datasets across the UK relevant to colorectal cancer and makes them available for research. As the four home nations (i.e. England, Scotland, Wales and Northern Ireland) have distinct data governance policies, data access and linkage has proven challenging. It has not yet been possible to create a UK-wide resource, hence limiting the potential of the data available. To address this, the Hub has recently sought to gain new governance approvals to link data from the four home nations. As part of this process, we aimed to investigate public acceptability of using confidential patient information (CPI) without consent in CORECT-R.

## Results

Seventy-five participants from a diverse background attended one of four online consultation sessions. Participants raised a number of issues and concerns, including ensuring transparency in the access and use of CPI by academic and commercial partners, and being well-informed of how to opt-out. With open communication and explanation of our approach, participants gave almost unanimous support on the intended CPI use.

## Implications

Innovation and collaboration between researchers, data custodians and the public are essential to drive responsible and meaningful uses of population data. We showed an exemplar of embedding patient and public involvement in designing data linkage research. Key areas identified from the sessions will form the basis of future consultations.

